# Ketone body 3-hydroxybutyrate as a biomarker of aggression

**DOI:** 10.1038/s41598-021-84635-6

**Published:** 2021-03-12

**Authors:** A. M. Whipp, E. Vuoksimaa, T. Korhonen, R. Pool, A. But, L. Ligthart, F. A. Hagenbeek, M. Bartels, L. H. Bogl, L. Pulkkinen, R. J. Rose, D. I. Boomsma, J. Kaprio

**Affiliations:** 1 Institute for Molecular Medicine Finland (FIMM), University of Helsinki, Helsinki, Finland; 2 Department of Biological Psychology, Vrije Universiteit Amsterdam, Amsterdam, The Netherlands; 3 Amsterdam Public Health (APH) Research Institute, Amsterdam, The Netherlands; 4 Clinicum, Department of Public Health, University of Helsinki, Helsinki, Finland; 5 Department of Epidemiology, Centre for Public Health, Medical University of Vienna, Vienna, Austria; 6 Department of Psychology, University of Jyvaskyla, Jyvaskyla, Finland; 7 Department of Psychological and Brain Sciences, Indiana University, Bloomington, Indiana USA

**Keywords:** Psychology, Biomarkers

## Abstract

Human aggression is a complex behaviour, the biological underpinnings of which remain poorly known. To gain insights into aggression biology, we studied relationships with aggression of 11 low-molecular-weight metabolites (amino acids, ketone bodies), processed using ^1^H nuclear magnetic resonance spectroscopy. We used a discovery sample of young adults and an independent adult replication sample. We studied 725 young adults from a population-based Finnish twin cohort born 1983–1987, with aggression levels rated in adolescence (ages 12, 14, 17) by multiple raters and blood plasma samples at age 22. Linear regression models specified metabolites as the response variable and aggression ratings as predictor variables, and included several potential confounders. All metabolites showed low correlations with aggression, with only one—3-hydroxybutyrate, a ketone body produced during fasting—showing significant (negative) associations with aggression. Effect sizes for different raters were generally similar in magnitude, while teacher-rated (age 12) and self-rated (age 14) aggression were both significant predictors of 3-hydroxybutyrate in multi-rater models. In an independent replication sample of 960 adults from the Netherlands Twin Register, higher aggression (self-rated) was also related to lower levels of 3-hydroxybutyrate. These exploratory epidemiologic results warrant further studies on the role of ketone metabolism in aggression.

## Introduction

Aggression is a significant behavioural problem affecting children and adults, with consequences not only for individuals, but families, schools, and communities as well^1^. It is also a complex and heterogenous behaviour that has been examined from multiple perspectives, including epidemiologic^2^, genetic^3^, epigenetic^4,5^, and biomarkers^6^, to name a few. With the heritability of aggression in both children and adults found to be about 50%^3^, aggression has a strong biological basis. Yet, much variation in aggressive behaviour remains to be explained. Some of the mechanisms, pathways, and molecules involved in the biological-level aspects of the behaviour are known; however, in both adults and children, there remain biological pathways to discover and biomarkers yet to identify^6–8^. With increased understanding of the biology of aggression, the opportunity to improve our interventions as well as pharmacological treatments arises. Thus, there is a need to generate new hypotheses and approaches to enhance our understanding of aggression and its associated problems.

One such suggested approach is using proton nuclear magnetic resonance (NMR) spectroscopy to find new biomarkers, because it allows for a hypothesis-free way to examine phenotypic associations^9–11^. NMR is not a new technology, but applying the approach to behavioural/psychological problems, including aggression, is gaining interest^6^. The NMR methodology is generally able to produce information on the levels of lipids, amino acids, organic acids, and other small organic molecules. Associations between lipid concentrations and aggression have previously been noted^6,12–15^, while other low-molecular-weight molecules might hold more promise for novel findings. Amino acids and ketone bodies (low-molecular-weight molecules categorized in NMR) have been associated with emotional and behavioural problems other than aggression^16–20^, and some studies have shown amino acids and similar molecules associated with anger and psychopathy^21^, schizophrenia-related violence^15^, and childhood aggression^22^. Accordingly, these areas offered a reasonable place to focus our exploratory investigation.

Thus, in this study we combine an NMR approach with the aggression phenotype in efforts to generate new insights into our biological understanding of aggression. We conducted exploratory analyses on the association between aggression and amino acid and ketone body biomarkers, seeking to explore association(s) found in more depth and with an attempt to replicate them in an independent sample. The discovery dataset includes longitudinal aggression measurements in adolescence and biomarker data in young adulthood, while the replication dataset includes biomarker and aggression data in young adults.

## Materials and methods

### Discovery dataset (FinnTwin12)

The FinnTwin12 study is a longitudinal population-based cohort of Finnish twins born in 1983–1987 that was initiated to track behavioural development and health habits from mid-childhood onward^23,24^. Twins and their families were identified through the Finnish Central Population Registry and initial enrolment of the twins occurred between the ages of 11 and 12. The baseline response rate was 87% (N = 5600 twins) and remained high (between 85 and 90%) for all data collection waves. Questionnaire data collection waves occurred around ages 11/12, 14, 17, and 22, and were collected from parents (age 11/12), teachers (age 11/12 and 14), and twins themselves (ages 14, 17 and 22).

Additionally, a subset of twins (from 1035 families) was more intensively studied using additional assessments (semi-structured psychiatric interview and questionnaires) in young adulthood (mean age = 22.4, SD = 0.70; n = 1347, response rate 73.0%), as well as a plasma sample, anthropometrics and neuropsychological tests for twins tested in person in young adulthood (n = 779). Previous investigation has shown that these more intensively studied twins do not differ from the rest of the cohort regarding behavioural or emotional problems^25^.

A blood sample was collected after an overnight fast with the request that the participant abstain from alcohol and tobacco since the night before the sample. Plasma was extracted immediately and stored at − 80 °C. Samples were processed in one batch in the autumn of 2010 using an automated high-throughput serum NMR metabolomics platform (i.e., the Brainshake/Nightingale platform)^24,26,27^. This platform has demonstrated good quality control related to sample preparation and experimental factors^28,29^. The metabolites utilized here were the amino acids (alanine, glutamine, histidine, isoleucine, leucine, phenylalanine, tyrosine, valine) and ketone bodies (acetate, acetoacetate, 3-hydroxybutyrate). All metabolite data were available with units mmol/l. Pregnant women (n = 53) and individuals on cholesterol medication (n = 1) were excluded from analysis; those excluded were no different in their aggression scores compared to those included. Thus, the final sample for study was 725 twin individuals.

Ethical approval for all waves of data collection was obtained from the ethical committee of the Helsinki and Uusimaa University Hospital District and Indiana University’s Institutional Review Board. All data collection and sampling was performed in compliance with the ethical guidelines. At ages 12 and 14, parents provided consent for the twins, while twins themselves provided written consent for age 17 and 22 collection waves.

#### Aggression measurement

Behavioural and emotional development in FinnTwin12 were measured at ages 12, 14, and 17 (from parents, teachers, twins themselves, and co-twins rated their twin as well) using the modified Multidimensional Peer Nomination Inventory (MPNI)^30^. The MPNI is a 37-item questionnaire producing multiple subscales including aggression (6 items; 4 direct aggression items, 2 indirect aggression items). Each question about the child’s behaviour (e.g., “Does the child call people names when angry with them?”) has four response choices: ‘not observed in child’, ‘observed sometimes, but not consistently’, ‘definitely observed, but is not prominent’, and ‘clearly observed’. Response choices are scored 0–3, and aggression scores for individual raters (e.g., teacher rating at age 12) are formed by taking the mean of all items in the aggression subscale (no missing values were allowed). A general aggression score was created (using all 6 items). We use the four different types of raters at three different ages (seven aggression ratings in total) because the literature indicates that different raters observe different aspects of children’s behaviour in the different settings available to them^31,32^, and by using multiple ratings we can assess the robustness of the biomarker associations across settings, observers, and adolescence. Additionally, a composite aggression score (called ‘Aggression Combined’) was used for initial analyses in order to minimize multiple testing and to combine information from different informants, who tend to rate aggression slightly differently^31,33^. It was formed by calculating the mean of the aggression subscale scores for the parent rating at age 12 and the teacher ratings at ages 12 and 14 combined.

#### Covariates

Based on careful literature review, several covariates, measured at the time of sample collection, were included in the regression model because they were related to both biomarker levels and aggression, and they were available in both the discovery and the replication samples. Body mass index (BMI) was calculated as kg/m^2^ (mean: 23.3, standard deviation (SD): 3.94). Leisure-time physical activity was expressed as metabolic equivalent of task (MET) hours/day based on structured questions considering the intensity, frequency, and duration of activities^34^. Smoking status was categorized as current, former, never/experimenter. Alcohol consumption frequency was categorized as none, once a month or less, 2–4 times per month, 2–3 times per week, 4 or more times per week. BMI, physical activity, alcohol use and smoking have been shown to be related to both metabolite levels^26,35–37^ and aggression^38–40^. Finally, self-rated general health was dichotomized to either poor health (poor + fair) or good health (good + very good). This measure was included to roughly capture somatic health problems (e.g., diabetes) in these young adults that could affect their metabolite levels^41^, and which could also be related to aggression^42^.

### Replication dataset (NTR)

The Netherlands Twin Register (NTR), established in 1987, collects data on twins and their family members. Data collection is nationwide via questionnaires mailed to parents of young twins (ages 1–12) and to twins themselves at age 14 and beyond. Twins recruited as adolescents and adults and their families are invited to take part in data collection by survey every 2–4 years^43^. Multiple survey waves have included the Achenbach System of Empirically Based Assessment (ASEBA) Adult Self-Report (ASR)^44^, which includes questions that allow for the calculation of an aggression score. A subgroup of adult participants was invited into the NTR Biobank project^45,46^. As part of this project, they were visited at home where samples were collected between 7:00 and 10:00 after overnight fasting. Participants were asked not to smoke before sampling. Fertile women took part from day 2–4 of their menstrual cycle, or in the pill-free week. During the visit, body composition measurements were taken and information regarding physical health and lifestyle behaviour (e.g., smoking and drinking patterns, exercise, medication use) was collected.

Metabolite data, processed using the same Brainshake/Nightingale platform as FinnTwin12^27^, were assessed in 5204 participants (age range: 18–87 years). As in FinnTwin12, we excluded women who were pregnant (n = 19); those excluded were no different in their aggression scores compared to included individuals. Additionally, all individuals over the age of 30 years at the time of sampling (n = 3843) were excluded to make the FinnTwin12 and NTR samples similar in age composition and because changes in body chemistry occur that accompany, for example, menopause and the ageing process for both sexes^47^. The two data collection timepoints (wave 8 and 10) that obtained aggression scores (ASR questionnaire) and were closest to the time of blood sampling were analysed. ASR aggression scores at wave 8 were available from 819 participants (mean age: 28.1 years, range 18–38 years), and ASR aggression scores from wave 10 from 665 participants (mean age: 32.7 years, range 21–39 years). The aggression score used for the analyses was the score closest to the time of blood sample collection. Of note, aggression scores were generally available closer to the time of sampling for the NTR dataset (mean 3.4 years; range 0–10 years) than the FinnTwin12. There were 150 (non-pregnant, age 30 or younger at time of blood sampling) participants who had aggression score values collected before their blood sample, and these scores were excluded to make the dataset temporally uniform (blood samples first, aggression scores later; this is the opposite of the temporality of the FinnTwin12 sample). Their aggression scores were no different than those of included individuals. Thus, the final sample for study was 960 individuals. For replication, only the metabolite 3-hydroxybutyrate (mmol/l) was examined.

NTR projects were approved by the Central Ethics Committee on Research Involving Human Subjects of the VU University Medical Centre, Amsterdam, an Institutional Review Board certified by the U.S. Office of Human Research Protections (IRB number IRB00002991 under Federal-wide Assurance FWA00017598; IRB/institute code NTR 03-180). All data collection and sampling were performed in compliance with the ethical guidelines, and all participants provided written informed consent.

#### Aggression measurement

Aggression in the Adult NTR was analysed from surveys 8 and 10, which both included the ASR, part of the ASEBA for assessing problems and strengths in children and adults, with multiple subscales including aggression^44^. Each aggression question has three response choices which were summed over items, if the number of missing items was not larger than three. For participants with no more than three missing values, missing items were imputed. If participants had completed the ASR at both surveys 8 and 10, we took their aggression score closest to the blood sampling (but always after blood sampling).

#### Covariates

We aimed to mirror variables and categories as close to the FinnTwin12 dataset as possible. BMI was calculated as kg/m^2^ (from the biobank home visit) (mean: 23.0, SD: 3.43). Leisure-time physical activity (from the same survey wave that the aggression score was taken) was calculated as MET minutes. Smoking status was categorized as current, former, never (from the biobank home visit). Alcohol consumption frequency (from the same survey wave as the aggression score) was categorized as none, once a month or less, 2–4 times per month, 2–3 times per week, 4 or more times per week. Self-rated general health (from the same survey wave that the aggression score was taken) was dichotomized to either poor health (poor + fair) or good health (reasonable + good + very good). Since the NTR metabolite data were processed in different batches, there was a batch variable available for adjustment.

### Analysis

For descriptive statistics (supplemental), we calculated means and standard deviations (SD) and ranges for the aggression scores for the different raters that were used in the analyses in FinnTwin12 (as well as in the NTR replication dataset).

Exploratory analyses in FinnTwin12 involving the available low-molecular-weight metabolites (n = 11; 8 amino acids and 3 ketone bodies) utilised the composite aggression score (Aggression Combined). Spearman correlations were computed between the untransformed metabolites, and between the metabolites and the Aggression Combined variable. Thereafter, due to their non-normal distribution, all metabolites were rank transformed (generally the best solution for most of the metabolites). Then, linear regression was performed with individual metabolites as the response variable and Aggression Combined as the main predictor variable, adjusted for age, sex, and BMI. To obtain robust standard errors (i.e., corrected for familial relatedness), the cluster option in Stata was specified^48^. Beta coefficients and 95% confidence intervals (CI) were reported and those reaching significance (p < 0.05) were investigated further. After exploratory analyses were performed, we proceeded to investigate significant findings further in an independent sample of young adults.

To further investigate 3-hydroxybutyrate (the only biomarker that reached a nominal p < 0.05 adjusted for multiple testing for 9 independent variables obtained from Matrix Spectral Decomposition (matSpD: https://gump.qimr.edu.au/general/daleN/matSpD/), which estimated the equivalent number of independent variables in our correlation matrix of 11 metabolites), additional linear regression modelling was employed to establish consistency of the association. In addition to the primary Aggression Combined variable, we examined the separate general aggression ratings from different raters as well (parents at age 12, teacher(s) at age 12, teacher(s) at age 14, self ratings at ages 14 and 17, and the twin’s co-twin at ages 14 and 17). Initial models designated 3-hydroxybutyrate as the response variable and the different general aggression ratings as the main predictor variable, adjusted for age, sex, BMI, and familial relatedness. Further models additionally adjusted for leisure-time physical activity (METs), smoking status, alcohol consumption frequency, and self-rated general health. Sex interactions were also investigated. Supplemental post hoc analysis investigated whether including multiple general aggression ratings in the same model could clarify the results, since a previous investigation of ours indicated that multiple raters in one model improved aggression information (different raters observe in different settings and can provide unique information^31^) and improved prediction of a later personality disorder^33^.

To obtain further evidence to support or refute the association found in our discovery sample, we analysed the NTR dataset to test replication of our results. The initial Spearman correlation of the untransformed 3-hydroxybutyrate and the aggression rating was computed. Then, the 3-hydroxybutyrate variable was rank transformed, as in the FinnTwin12 data. Linear regression models were run with 3-hydroxybutyrate as the response variable and the aggression rating as the predictor variable, adjusted for age, sex, BMI, batch and familial relatedness. Additionally, fully adjusted models further adjusted for leisure-time physical activity (METs), smoking status, alcohol consumption frequency, and self-rated general health. Sex interactions were also investigated to study if the aggression–3-hydroxybutyrate relationship differed in males and females^49^.

Finally, additional analyses were run to verify the maximum likelihood estimates of the association examined in the fully adjusted frequentist models above with a Bayesian approach, a powerful tool for incorporating information from previous studies, for controlling confounding, for interpretation of study results and evaluation of hypotheses about exposure–disease relationships^50^. We fitted several Bayesian random-intercept (2-level or mixed) regression models separately on the FinnTwin12 and NTR data with 3-hydroxybutyrate as the response variable and the aggression rating as the predictor variable (we used ‘Aggression Combined’ for FinnTwin12). We adjusted for age, sex, BMI, MET, smoking status, alcohol consumption frequency, and self-rated general health (batch was also included for NTR), and ran Bayesian models with and without an interaction of sex and aggression included, as well as stratified on sex. The clustering of the twins was accounted for by setting each twin pair a random intercept.

The Bayesian model parameters to estimate included the overall intercept, the regression coefficients and the random intercepts along with their variance components. For the overall intercept and the regression coefficients, we set a weakly informative (default) prior (normal (0,10,000)). For the variance of the error term, we set a non-informative (default) prior (inverse-gamma prior with shape and scale parameters equal to 0.1). For the random intercepts, we set a hierarchical prior normal (0, sigma), with the hyperparameter sigma set a uniform prior with bounds at the 95% confidence interval of the maximum likelihood estimate of the variance of the random intercepts (i.e., 95% CI for the variance from the frequentist random-intercept model). In fact, there appeared a convergence problem when using a non-informative (default) prior for the variance of the random intercepts. To overcome this problem, we incorporated information on the probable range of the variance via the prior distribution. Gibbs sampling was used to draw samples from the posterior distribution (20,000 iterations, including a burn-in of 7000 iterations). To confirm the convergence of Markov chain Monte Carlo (MCMC) chains, we performed a visual evaluation (trace, density and autocorrelation plots). From these models, we reported the estimated regression coefficient for aggression as the posterior expectation (mean) and 95% credible intervals assessed from the posterior distribution. Lastly, to assess whether the use of informative priors affects the results, we performed a sensitivity analysis by incorporating information on the association obtained from the Bayesian sex-stratified analysis of the FinnTwin12 cohort to the priors for the aggression coefficients in the NTR sex-stratified models. The coefficients were assigned the normal distribution with the mean set to the posterior mean and the variance set to the squared standard deviation obtained from the corresponding Bayesian sex-stratified analysis on the FinnTwin12 cohort.

All analyses were performed using Stata version 15.1 or 16.0 (Stata Corporation, College Station, TX, USA).

## Results

### Discovery (FinnTwin12) results

Descriptive means (SD) and ranges of the aggression scores from the different raters used in the analyses are provided in Supplemental Table 1. Spearman correlations between untransformed metabolites indicated generally low to moderate associations, except the high correlations between 3-hydroxybutyrate and acetoacetate (r = 0.70) and the branched-chain amino acids (leucine, isoleucine, and valine) with each other (r range = 0.62–0.73) (Table 1). Spearman correlations between metabolites and the Aggression Combined variable were low, ranging from − 0.13 to 0.14.

**Table 1:** FinnTwin12 Spearman correlations of untransformed metabolites and aggression (n=526).

	ace	acace	bohbut	ala	gln	his	ile	leu	phe	tyr	val
acace	0.11										
bohbut	0.15	0.70									
ala	−0.12	−0.38	−0.36								
gln	0.36	−0.03	−0.05	0.06							
his	0.07	−0.01	−0.11	0.20	0.00						
ile	0.11	0.22	−0.07	0.19	0.28	0.09					
leu	0.12	0.13	−0.04	0.08	0.17	0.02	0.73				
phe	−0.06	−0.03	−0.05	0.16	−0.32	0.30	0.15	0.34			
tyr	0.16	−0.12	−0.23	0.38	0.39	0.20	0.42	0.30	0.15		
val	0.20	0.16	−0.02	0.07	0.37	0.05	0.68	0.62	0.03	0.55	
AggComb^a^	−0.04	0.04	−0.13	0.04	0.09	0.07	0.14	0.10	−0.06	0.13	0.11

Abbreviations: *ace* acetate, *acace* acetoacetate, *AggComb* aggression combined, *bohbut* 3-hydroxybutyrate, *ala* alanine, *gln* glutamine, *his* histidine, *ile* isoleucine, *leu* leucine, *phe* phenylalanine, *tyr* tyrosine, *val* valine.

^a^Aggression is 'Aggression Combined' variable (which is the mean of parent-12, teacher-12, and teacher-14 aggression ratings).

**Figure 1:**
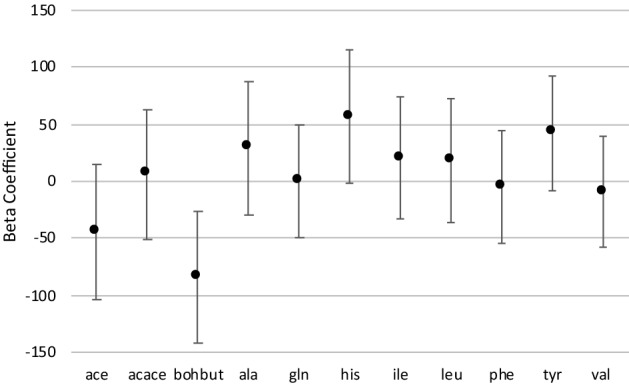
FinnTwin12 regression coefficients (and 95% confidence intervals) of Aggression Combined predictor variable in linear regression models with metabolites as response variables, adjusted for age, sex, body mass index, and familial relatedness. *ace *acetate, *acace *acetoacetate, *bohbut* 3-hydroxybutyrate, *ala* alanine, *gln* glutamine, *his* histidine, *ile* isoleucine, *leu* leucine, *phe* phenylalanine, *tyr* tyrosine, *val* valine.

Next, linear regression models with rank-transformed metabolites as the response variable and Aggression Combined as the main predictor variable, also adjusted for age, sex, BMI, and familial relatedness, revealed that only 3-hydroxybutyrate was significantly associated with aggression, including after adjustment for multiple testing (Fig. 1; Supplemental Table 2). The association was negative, with lower 3-hydroxybutyrate levels being associated with higher aggression levels.

**Figure 2:**
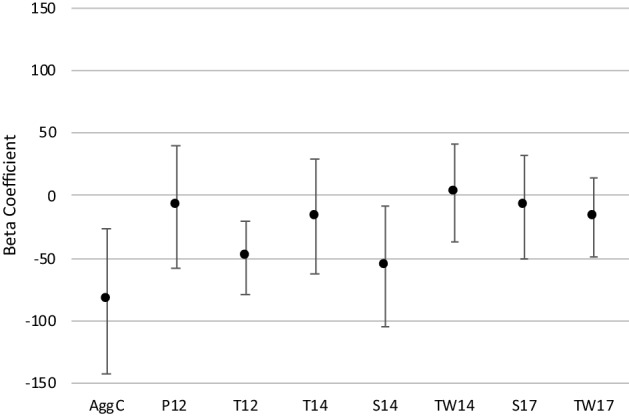
FinnTwin12 regression coefficients (and 95% confidence intervals) of aggression ratings from different raters as (separate) predictor variables in linear regression models with rank-transformed 3-hydroxybutyrate as the response variable, adjusted for age, sex, body mass index, and familial relatedness. *AggC* aggression combined, *P12* parent rating age 12, *T12* teacher rating age 12, *T14* teacher rating age 14, *S14* self rating at age 14, *TW14* co-twin rating at age 14, *S17* self rating at age 17, *TW17* co-twin rating at age 17.

In linear regression models further exploring 3-hydroxybutyrate’s association with aggression, the basic model (3-hydroxybutyrate as the response variable, and aggression ratings from different raters as main predictor variables in separate models, adjusted for age, sex, BMI, and familial relatedness) results indicated that nearly all aggression ratings demonstrated the trend of negative association of 3-hydroxybutyrate with aggression, with teacher ratings at age 12 and self ratings at age 14 showing the strongest effect sizes (and also being statistically significant) (Fig. 2; Supplemental Table 3). In fully adjusted models (basic model plus MET, smoking status, alcohol consumption frequency, and self-rated general health), the negative associations remained, with only small attenuation to the effect sizes (Table 2). No sex interactions were detected (p-value range = 0.10–0.84; Supplemental Table 4).

**Table 2:** FinnTwin12 multivariable linear regression models with 3-hydroxybutyrate as the response variable, regression coefficients of aggression ratings from different raters as predictor variables in separate models, adjusted for age, sex, BMI, MET, smoking status, alcohol consumption frequency, self-rated general health, and familial relatedness.

Aggression rating	Co-variates	N	Unstandardised Beta Coeff (95% CI)	Standardised Beta Coeff (p-value)	R-squared^a^
AggCombined	Age, sex, BMI*, MET, smoking, alcohol, health	524	− 84.1 (− 143.9, − 24.4)	− 0.13 (0.006)	0.042
P12	Age, sex, BMI*, MET, smoking, alcohol, health	677	− 4.5 (− 54.0, 45.0)	− 0.08 (0.9)	0.024
T12	Age, sex, BMI*, MET, smoking, alcohol, health	688	− 49.1 (− 78.4, − 19.9)	− 0.14 (0.001)	0.039
T14	Age, sex, BMI*, MET, smoking, alcohol, health	553	− 13.3 (− 60.3, 33.7)	− 0.03 (0.6)	0.023
S14	Age, sex, BMI*, MET, smoking, alcohol, health	684	− 56.2 (− 105.2, − 7.2)	− 0.09 (0.02)	0.030
TW14	Age, sex, BMI*, MET, smoking, alcohol, health	682	2.2 (− 38.2, 42.6)	0.004 (0.9)	0.022
S17	Age, sex, BMI*, MET, smoking, alcohol, health	639	− 11.3 (− 53.5, 31.0)	− 0.02 (0.6)	0.018
TW17	Age, sex, BMI, MET, smoking, alcohol, health	632	− 15.8 (− 49.0, 17.4)	− 0.04 (0.3)	0.018

*AggCombined *aggression combined, *BMI *body mass index, *CI *confidence interval, *MET *metabolic equivalent of task, *P12* parent rating age 12, *S14* self rating at age 14, *S17 *self rating at age 17, *T12 *teacher rating age 12, *T14 *teacher rating age 14, *TW14 *co-twin rating at age 14, *TW17 *co-twin rating at age 17.

*This co-variate also significant (p < 0.05).

^a^R-squared represents the variation explained from all variables in the model together.

In supplemental analyses, because the aggression scores from the different raters were only weakly to moderately correlated (r range: 0.14–0.47), multiple aggression ratings were added into the same model as predictor variables, adjusted for age, sex, BMI, and familial relatedness. The tested combinations included the three aggression scores that made up the Aggression Combined variable (parent age 12, teacher age 12, teacher age 14), both age 12 ratings, all three age 14 ratings, and both age 17 ratings, as well as the two ratings that were consistently significant (teacher age 12 and self age 14). Interestingly, in the model including teacher age 12 and self age 14 ratings, both were statistically significantly negatively associated with 3-hydroxybutyrate (Supplemental Table 5).

### Replication (NTR) results

In the NTR replication dataset, the Spearman correlation between the aggression score and untransformed 3-hydroxybutyrate was − 0.07 (p = 0.045). The initial linear regression model with rank-transformed 3-hydroxybutyrate as the response variable and aggression as the main predictor variable, adjusted for age, sex, BMI, batch and familial relatedness, showed the same negative association trend (p = 0.138) as the FinnTwin12 models (Table 3). There was no sex interaction detected in the initial model (p = 0.246), however, in the fully adjusted model there was (p = 0.015). Regarding the fully adjusted models, the negative association was present for males only; it became slightly positive in direction for females, albeit with wide confidence intervals. Table 3 displays results for the sexes pooled and males and females modelled separately.

**Table 3:** NTR multivariable linear regression models (initial and fully adjusted^a^) with 3-hydroxybutyrate as the response variable, regression coefficients of aggression rating as predictor variable, with sexes pooled and separated.

Model	Co-variates	N	Unstandardised Beta Coeff (95% CI)	Standardised Beta Coeff (p-value)	R-squared^b^
**Initial model**^a^					
Both sexes	Age, sex*, BMI, batch*	811	− 22.6 (− 52.5, 7.3)	− 0.05 (0.1)	0.151
Male	Age, BMI, batch*	253	− 48.8 (− 118.7, 21.2)	− 0.09 (0.2)	0.221
Female	Age, BMI, batch*	558	− 14.1 (− 47.6, 19.5)	− 0.03 (0.4)	0.108
**Fully adjusted model**^a^					
Both Sexes	Age, sex*, BMI, batch*, MET, smoking, alcohol*, health	567	− 11.9 (− 48.4, 24.7)	− 0.03 (0.5)	0.153
Male	Age, BMI, batch*, MET*, smoking, alcohol, health	165	− 85.5 (− 171.8, 0.8)	− 0.14 (0.052)	0.270
Female	Age, BMI, batch*, MET, smoking, alcohol*, health	402	11.8 (− 28.4, 52.1)	0.03 (0.6)	0.102

^a^Initial model: adjusted for age, sex, BMI, batch, and familial relatedness. Fully adjusted model: adjusted for age, sex, BMI, batch, MET, smoking status, alcohol consumption frequency, self-rated general health, and familial relatedness.

^b^R-squared represents the variation explained from all variables in the model together.

### Bayesian modelling

For all the fitted Bayesian models, the convergence checks revealed no autocorrelation and demonstrated the MCMC achieved the target distribution. In FinnTwin12, the initial non-informative priors model without the sex interaction term resulted in an aggression rating variable posterior mean of − 84.0 (95% credible interval: − 91.0, − 76.9), while the model with the sex × aggression interaction term indicated a posterior mean of − 86.6 (95% credible interval: − 100.4, − 72.5) for the interaction term. For the males-only model, the aggression posterior mean was − 37.9 (95% credible interval: − 43.8, − 32.0). For the females-only model, the aggression posterior mean was − 119.5 (95% credible interval: − 127.8, − 111.1). In NTR, the initial non-informative priors model without the sex interaction term resulted in an aggression rating variable posterior mean of 10.6 (95% credible interval: − 15.3, − 5.9), while the model with the sex × aggression interaction term indicated a posterior mean of 83.5 (95% credible interval: 72.5, 94.4) for the interaction term. For the males-only model, the aggression posterior mean was − 80.4 (95% credible interval: − 86.3, − 74.6). For the females-only model, the aggression posterior mean was 14.2 (95% credible interval: 9.9, 18.5). Finally, in the sensitivity analysis of NTR sex-stratified models using (FinnTwin12-based) informative priors for the sex-specific regression coefficients for aggression, the results remained similar (data not shown).

## Discussion

In this study, we initially tested 11 potential NMR-processed low-molecular-weight biomarkers for association with a composite aggression score, and 3-hydroxybutyrate, a ketone body, was the only nominally statistically significant biomarker (negatively associated with aggression). Upon further analysis, in both basic and fully adjusted models, nearly all aggression ratings (from different raters and ages) showed a consistent pattern of negative association, with three being statistically significant. When including both teacher (age 12) and self (age 14) aggression ratings in the same model, both were significantly associated with 3-hydroxybutyrate, showing that an external rater (teacher) and an adolescent’s self-rating contribute uniquely to this association. Importantly, we were able to assess this biomarker–aggression association in a large and independent sample, the NTR. In both basic and fully adjusted NTR (frequentist) models, the negative association of 3-hydroxybutyrate and aggression was found, but statistical significance was not reached until the sexes were separated in the fully adjusted model (as indicated by the significant sex interaction). Finally, Bayesian modelling indicated a sex interaction in both FinnTwin12 and NTR cohorts, and the negative association—low 3-hydroxybutyrate levels with high aggression levels—remained in sex-stratified models (with non-informative priors) for both sexes in the FinnTwin12 cohort and for males only in the NTR cohort.

The association of 3-hydroxybutyrate with aggression is unlikely to be spurious because it was seen in two independent samples, and was consistent across raters and ages. Because we were not testing any specific hypothesis, and thus were not guided solely by statistical significance (p-values), our focus was on identifying repeated patterns of associations, which we saw here. Additionally, although we were not addressing causality in these models, we saw stability in the trend regardless of temporality (biomarker measured before or after aggression). Sex differences were evident, but these will need to be clarified by future investigations. Furthermore, the trend in FinnTwin12 was nearly uniform across raters and ages. This suggests that the association is related to generalized aggression (liability to behave aggressively in different contexts), which is a more serious and important type of aggression because it leads to the poorest outcomes^51^. Our model including multiple raters of aggression in the same model also supports this notion of generalized aggression, with ratings from both the teachers and adolescents themselves uniquely contributing to the association of aggression with 3-hydroxybutyrate. The teen him/herself provides their perspective about their behavior across all environments, while teachers have a unique perspective. Compared to parents for example, teachers regularly observe the child amongst other children of the same age, making for more valid predictions of the student’s liability to behave aggressively^33,51^. Combining both of these types of observers provides unique information, helping to clarify the breadth of this association of aggression with 3-hydroxybutyrate.

To our knowledge, this is the first time ketone bodies, and 3-hydroxybutyrate specifically, have been associated with aggression. In a urinary metabolite study with aggression in the NTR, no significant associations were found, but several near-significant results pointed to energy metabolism as a potential group of metabolites warranting further investigation^22^. Additionally, there are two recent studies showing a link between 3-hydroxybutyrate and depression, one in mice that was negatively correlated^18^ and one in humans that was positively correlated^19^. We know from previous studies that depression and aggression often co-occur^25,52^, and the concept of a ‘p factor’ that potentially underlies all psychopathology has been gaining traction^53^. However, our finding of a negative aggression–3-hydroxybutyrate relationship is in contrast with earlier reported positive depression–3-hydroxybutyrate relationships and warrants studying these traits separately in relation to metabolites.

In evaluating the biological plausibility of this association, we can consider generally what we know about this compound and how it might relate to aggression. During periods of exercise or fasting (low availability of glucose as an energy source), ketone bodies become involved in an alternative energy pathway^41^. 3-Hydroxybutyrate, in particular, has been shown to be not only involved in the alternative energy pathway, but it also has important cell signalling and regulatory functions, including pathways involved in reducing oxidative stress and inflammation (both linked to aggression^7,54^) and epigenetic modifications^19,55^. We also know that while 3-hydroxybutyrate is generally produced in the liver, it can cross the blood–brain barrier and act in the brain^56,57^, where we know that aggression is also regulated^58,59^. Finally, although there appear to be complex biological mechanisms at play, ketogenic diets (which increase ketone body levels) have been popular treatments in neurologic disorders, such as epilepsy, for decades^60^. The field of psychiatry has recently become more interested in the potential benefits of this diet, including in attention-deficit disorders and autism^61^, both of which can co-occur with aggression, however, aggression and the ketogenic diet has yet to be considered. Intriguingly, researchers have shown that a ketogenic diet with beta-hydroxybutyrate for male Drosophila with aggression after traumatic brain injury markedly reduced the levels of aggression^62^.

This study suggesting a new biomarker of aggression has important strengths in its large sample size, multiple ratings of aggression across stages of development, and the direction of association replicated in an independent sample. There are also limitations. First, we did not have aggression measured at the same time as the biological sample, in either dataset. However, it is known that aggression is a relatively stable trait, especially in aggressive males^51^. Although aggression levels in childhood and adolescence are generally higher than in adulthood, aggression levels are relatively stable in their rank order among similar aged peers^63,64^. There is longitudinal continuity in aggression from adolescence to adulthood^33,51,65^, which is the development period covered in the present study. Additionally, a fairly recent study with similar temporal data collection limitations, using childhood aggression measures (as developmental trajectories) and biological samples in young adulthood, was able to identify cytokines (a measure of inflammation) as a novel group of biomarkers for aggression^7^. Regarding biomarker stability, while we do see high heritability in the energy metabolism biomarkers (53.2% for 3-hydroxybutyrate)^66^, it would also be important in future studies to have repeated biological samples to confirm the stability of this biomarker over time, concurrent with the stability of aggression measures. However, repeated blood samples from children may be challenging to obtain, and thus this work might need to be done in young adults first, or using less invasive samples such as urine or saliva, which might produce different results than plasma (the recent urine biomarker study on NTR children did not show any significant findings with 3-hydroxybutyric acid and aggression)^22^.

In conclusion, this exploratory study using NMR-processed metabolites in plasma samples has revealed a potential new biomarker of aggression: 3-hydroxybutyrate, a ketone body related to an alternative energy pathway. We observed a negative association—high aggression levels with low ketone bodies levels—in our Finnish dataset, which was suggestively replicated in our independent Dutch dataset. There is biological plausibility for associating this ketone body with aggression, as well as the ability to potentially influence the biological levels of 3-hydroxybutyrate via diet modification. Thus, it is well-warranted to further investigate this epidemiological association at the biological level, hopefully shedding more light on the complexities and heterogeneity of the aggression phenotype.

## Supplementary Information

Supplementary Tables.

## Data Availability

The datasets generated and/or analysed during the current study are not publicly available due to participant confidentiality, but are available from the corresponding author on reasonable request.
