# Supplementary material for: Ketone body 3-hydroxybutyrate as a biomarker of aggression
Source: Sci Rep. 2021 Mar 12;11:5813. doi: 10.1038/s41598-021-84635-6 (PMC7955062; doi:10.1038/s41598-021-84635-6)
Supplement: Supplementary file 1 — Supplementary Tables. [file 41598_2021_84635_MOESM1_ESM.docx]

**Ketone body 3-hydroxybutyrate as a biomarker of aggression**

AM Whipp^1*^, E Vuoksimaa^1^, T Korhonen^1^, R Pool^2,3^, A But^4^, L Ligthart^2^, FA Hagenbeek^2,3^, M Bartels^2,3^, LH Bogl^1,5^, L Pulkkinen^6^, RJ Rose^7^, DI Boomsma^2,3^, J Kaprio^1,4^

**Supplemental Tables**

**Supp Table 1**. Means (standard deviations; SD) and ranges of aggression scores by rater for FinnTwin12 and NTR cohorts

| **Cohort (Instrument)** | **Rater (child age)** | **N** | **Mean (SD)** | **Range** |
| --- | --- | --- | --- | --- |
| FT12 (MPNI) | Parent (12) | 699 | 0.56 (0.39) | 0–2.33 |
|  | Teacher (12) | 710 | 0.60 (0.61) | 0–3 |
|  | Teacher (14) | 570 | 0.29 (0.44) | 0–2.67 |
|  | Self (14) | 706 | 0.46 (0.36) | 0–1.83 |
|  | Co-twin (14) | 703 | 0.56 (0.48) | 0–2.83 |
|  | Self (17) | 659 | 0.83 (0.43) | 0–2.29 |
|  | Co-twin (17) | 653 | 0.97 (0.55) | 0–2.86 |
| NTR (ASR) | Self (wave 8) | 819 | 3.04 (3.17) | 0–19 |
|  | Self (wave 10) | 665 | 2.98 (3.12) | 0–21 |

ASR: Adult Self-Report; MPNI: Multidimensional Peer Nomination Inventory

**Supp Table 2**. FinnTwin12 linear regression models with metabolites as response variables, regression coefficients of Aggression Combined^a^ as the predictor variable, adjusted for age, sex, BMI, and familial relatedness.

| **Biomarker^b^** | **Co-variates** | **N** | **Unstandardized**  **Beta Coeff**  **(95% CI)** | **Standardized**  **Beta Coeff**  **(p-value)** | **R-squared^c^** |
| --- | --- | --- | --- | --- | --- |
| ace | age, sex*, BMI | 537 | -44.53 (-103.58, 14.53) | -0.07 (0.1) | 0.059 |
| acace | age*, sex*, BMI | 536 | 5.65 (-51.06, 62.37) | 0.009 (0.8) | 0.072 |
| bohbut | age, sex, BMI* | 527 | -84.52 (-142.76, -26.28) | -0.13 (0.004) | 0.040 |
| ala | age, sex, BMI* | 537 | 29.33 (-29.14, 87.79) | 0.05 (0.3) | 0.033 |
| gln | age*, sex*, BMI | 537 | 0.35 (-49.31, 50.00) | 0.0005 (1.0) | 0.316 |
| his | age, sex, BMI | 537 | 56.07 (-2.43, 114.58) | 0.09 (0.06) | 0.016 |
| ile | age, sex*, BMI* | 535 | 20.12 (-33.11, 73.35) | 0.03 (0.5) | 0.314 |
| leu | age*, sex*, BMI* | 535 | 17.44 (-37.24, 72.12) | 0.03 (0.5) | 0.174 |
| phe | age, sex*, BMI* | 537 | -5.35 (-54.35, 43.66) | -0.008 (0.8) | 0.157 |
| tyr | age, sex*, BMI* | 532 | 42.63 (-7.79, 93.06) | 0.06 (0.1) | 0.188 |
| val | age, sex*, BMI* | 531 | -9.58 (-58.55, 39.39) | -0.01 (0.7) | 0.383 |

Abbreviations: ace=acetate; acace=acetoacetate; AggC=Aggression Combined; ala=alanine; BMI=body mass index; bohbut=3-hydroxybutyrate; CI=confidence interval; gln=glutamine; his=histidine; ile=isoleucine; leu=leucine; phe=phenylalanine; tyr=tyrosine; val=valine

*this co-variate also significant (p<0.05)

^a^combines P12, T12, T14 aggression ratings into one variable (mean of all three)

^b^biomarkers are all rank transformed

^c^R-squared represents the variation explained from all variables in the model together

**Supp Table 3**. FinnTwin12 linear regression models with 3-hydroxybutyrate as the response variable, regression coefficients of aggression ratings as predictor variables in separate models, adjusted for age, sex, BMI, and familial relatedness.

| **Aggression rating** | **Co-variates** | **N** | **Unstandardized**  **Beta Coeff (95% CI)** | **Standardized**  **Beta Coeff (p-value)** | **R-squared^a^** |
| --- | --- | --- | --- | --- | --- |
| AggCombined | age, sex, BMI* | 527 | -84.5 (-142.8, -26.3) | -0.13 (0.004) | 0.040 |
| P12 | age, sex, BMI* | 682 | -9.1 (-57.7, 39.6) | -0.02 (0.7) | 0.022 |
| T12 | age, sex, BMI* | 693 | -49.9 (-78.7, -21.1) | -0.14 (0.001) | 0.039 |
| T14 | age, sex, BMI* | 556 | -17.0 (-63.3, 29.2) | -0.03 (0.5) | 0.022 |
| S14 | age, sex, BMI* | 689 | -56.4 (-104.6, -8.1) | -0.09 (0.02) | 0.029 |
| TW14 | age, sex, BMI* | 687 | 2.7 (-36.4, 41.8) | 0.006 (0.9) | 0.021 |
| S17 | age, sex, BMI* | 644 | -9.2 (-50.3, 32.0) | -0.02 (0.7) | 0.018 |
| TW17 | age, sex, BMI | 637 | -17.6 (-49.5, 14.4) | -0.04 (0.3) | 0.017 |

Abbreviations: AggCombined=aggression combined; BMI=body mass index; CI=confidence interval; P12=parent rating age 12; S14=self rating at age 14; S17=self rating at age 17; T12=teacher rating age 12, T14=teacher rating age 14, TW14=co-twin rating at age 14, TW17=co-twin rating at age 17

^a^R-squared represents the variation explained from all variables in the model together

**Supp Table 4**. FinnTwin12 linear regression models (initial and fully adjusted^a^) with 3-hydroxybutyrate as the response variable, regression coefficients of Aggression Combined as the predictor variable, with sexes separated (to allow for comparison to NTR data in Table 3)

| **Model** | **Co-variates** | **N** | **Unstandardized**  **Beta Coeff (95% CI)** | **Standardized**  **Beta Coeff (p-value)** | **R-squared^b^** |
| --- | --- | --- | --- | --- | --- |
| Initial Model^a^ |  |  |  |  |  |
| Male | age, BMI | 224 | -37.3 (-114.0, 39.5) | -0.07 (0.3) | 0.035 |
| Female | age, BMI | 303 | -125.3 (-211.4, -39.3) | -0.16 (0.004) | 0.080 |
|  |  |  |  |  |  |
| Fully Adjusted Model^a^ |  |  |  |  |  |
| Male | age, BMI, MET, smoking, alcohol, health | 224 | -37.3 (-115.9, 41.4) | -0.07 (0.4) | 0.047 |
| Female | age, BMI*, MET, smoking, alcohol, health | 300 | -120.1 (-210.0, -30.2) | -0.15 (0.009) | 0.082 |

^a^Initial model: adjusted for age, BMI, and familial relatedness. Fully adjusted model: adjusted for age, BMI, MET, smoking status, alcohol consumption frequency, self-rated general health, and familial relatedness

^b^R-squared represents the variation explained from all variables in the model together

**Supp Table 5**. FinnTwin12 linear regression models with 3-hydroxybutyrate as the response variable, regression coefficients of aggression ratings as predictor variables in combined models, adjusted for age, sex, BMI, and familial relatedness.

| **Model** | **Co-variates** | **N** | **Unstandardized**  **Beta Coeff (95% CI)** | **Standardized**  **Beta Coeff (p-value)** | **R-squared^a^** |
| --- | --- | --- | --- | --- | --- |
| P12 + T12 + T14^b^ | age, sex, BMI* | 525 |  |  | 0.047 |
| P12 |  |  | 0.6 (-55.7, 56.9) | 0.001 (1.0) |  |
| T12 |  |  | -57.4 (-91.9, -22.9) | -0.16 (0.001) |  |
| T14 |  |  | 2.2 (-45.8, 50.2) | 0.004 (0.9) |  |
| P12 + T12 | age, sex, BMI* | 668 |  |  | 0.041 |
| P12 |  |  | -3.3 (-52.6, 46.1) | -0.006 (0.9) |  |
| T12 |  |  | -50.4 (-81.0, -19.8) | -0.14 (0.001) |  |
| T14 + S14 + TW14 | age, sex, BMI* | 531 |  |  | 0.030 |
| T14 |  |  | -7.6 (-56.7, 41.4) | -0.02 (0.8) |  |
| S14 |  |  | -53.8 (-109.5, 1.9) | -0.08 (0.06) |  |
| TW14 |  |  | 3.0 (-41.2, 47.2) | 0.006 (0.9) |  |
| S17 + TW17 | age, sex, BMI* | 629 |  |  | 0.018 |
| S17 |  |  | 0.8 (-42.4, 43.9) | 0.002 (1.0) |  |
| TW17 |  |  | -17.6 (-51.1, 15.8) | -0.04 (0.3) |  |
| T12 + S14 | age, sex, BMI* | 674 |  |  | 0.045 |
| T12 |  |  | -46.1 (-75.5, -16.6) | -0.13 (0.002) |  |
| S14 |  |  | -50.1 (-98.2, -2.0) | -0.08 (0.04) |  |

Abbreviations: AggCombined=aggression combined; BMI=body mass index; CI=confidence interval; P12=parent rating age 12; S14=self rating at age 14; S17=self rating at age 17; T12=teacher rating age 12, T14=teacher rating age 14, TW14=co-twin rating at age 14, TW17=co-twin rating at age 17

*means this co-variate was also significant

^a^R-squared represents the variation explained from all variables in the model together

^b^these are the 3 variables that combine to create the Aggression Combined variable
